# Supplementary figures and images for: Targeted transcript quantification in single disseminated cancer cells after whole transcriptome amplification
Source: PLoS One. 2019 Aug 20;14(8):e0216442. doi: 10.1371/journal.pone.0216442 (PMC6701776; doi:10.1371/journal.pone.0216442)

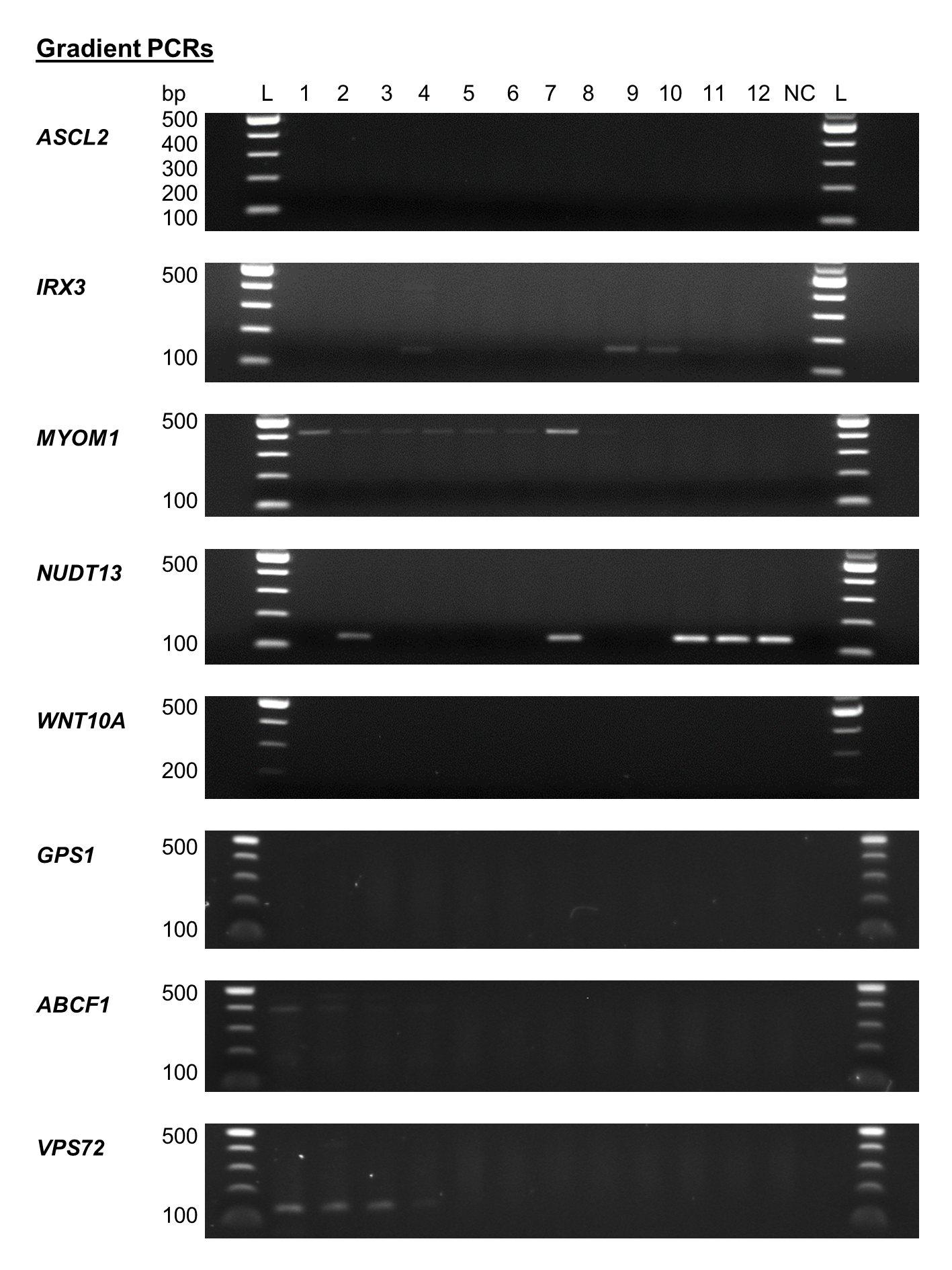

Supplement: S1 Fig — Gradient PCRs using different annealing temperatures (1–12: 53°C; 53.3°C; 54°C; 54.8°C; 56.1°C; 57.7°C; 59.6°C; 61.2°C; 62.3°C; 63.2°C; 63.8°C; 64°C); NC = Negative Control; L = 2-log DNA ladder. Template: reference cDNA. (TIF) [file pone.0216442.s001.tif]

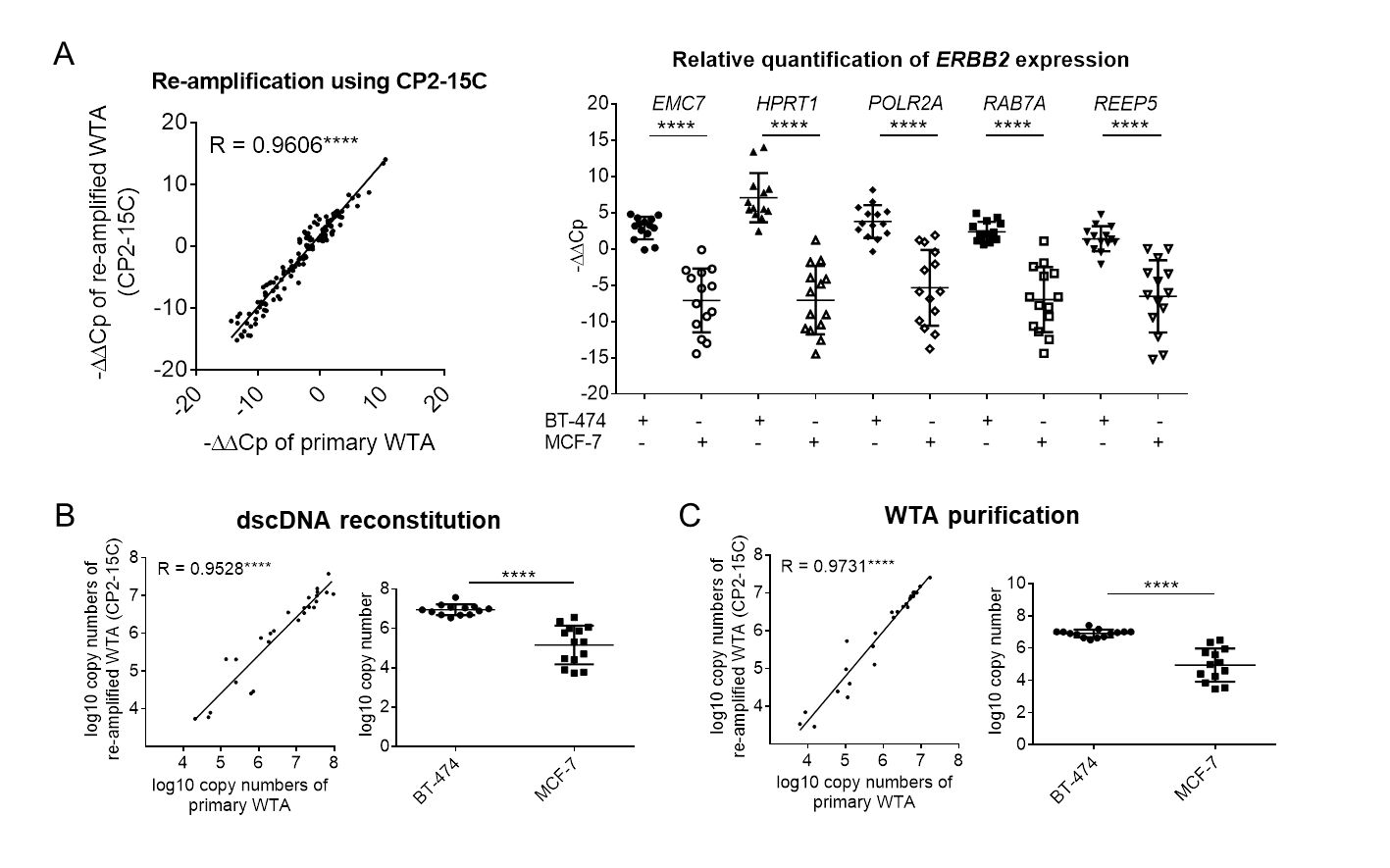

Supplement: S2 Fig — (A) Relative quantification analysis. Correlation between log2-transformed ratios (-ΔΔCp) of re-amplified compared to primary WTA products (left panel). Spearman’s correlation coefficient R. The right panel shows the relative quantification of ERBB2 expression at the single-cell level within BT-474 and MCF-7 using single reference genes as indicated. -ΔΔCp were calculated for every single cell, mean ± SD. (B,C: left panels) Correlation of ERBB2 qPCR results obtained by the absolute quantification strategy in re-amplified BT-474 and MCF-7 single cells between dscDNA reconstitution (B) or purified WTA (C) and diluted primary WTA products. Pearson’s correlation coefficients R. (B,C: right panels) Significant discrimination between BT-474 and MCF-7 cells by ERBB2 gene expression levels. Cp values were converted to log10 copy numbers using an external standard curve. Mean ± SD; Unpaired t-test with Welch’s correction; **** p<0.0001. (TIF) [file pone.0216442.s002.tif]
